# A systematic approach towards implementing value-based health care in heart failure: Understandings from retrospective analysis methods in South London

**DOI:** 10.1177/0951484820971442

**Published:** 2020-11-30

**Authors:** Emma Burnhope, Michael Waring, Andrew Guilder, Bharti Malhotra, Jorge M Cardoso, Reza Razavi, Gerald Carr-White

**Affiliations:** 1 Department of Cardiology, Guy’s and St Thomas’ NHS Foundation Trust, London, UK; 2 School of Biomedical Engineering and Imaging Sciences, King’s College London, London, UK; 3 Department of Radiology, Guy’s and St Thomas’ NHS Foundation Trust, London, UK

**Keywords:** information systems, methodology, value based healthcare

## Abstract

**Background:**

Value-Based Health Care (VBHC) is an evolving model of healthcare delivery aimed at achieving better patient outcomes at lower costs to the healthcare provider. The practise of VBHC requires efficient information systems with good reporting capability and subsequent outcome measuring. Information systems within the National Health Service (NHS) are often multiple and not necessarily integrated to one another. We therefore developed a systematic approach to collecting, validating and analysing data from multiple sources and information systems, with the aim of designing and endorsing an automatic system to capture health outcomes data in heart failure to support future VBHC models.

**Methods:**

A retrospective cohort of heart failure patients with reduced ejection fraction undergoing Implantable Cardioverter Defibrillator (ICD) or Cardiac Resynchronization Therapy (CRT) procedures within a limited geographical area in South London were evaluated. A purpose built database was created to integrate, transform and validate health care data from multiple information systems.

**Results:**

Validation analysis shows that our implemented methodology has produced a robust dataset. Our limited cohort of 134 patients does not allow for any complex statistical analysis however has identified some important themes related to outcomes and costs.

**Conclusion:**

We have created a validated database specific to our Trust that can be upscaled locally with ease and transferred to other health diseases. Due to variations in local procedure from one Trust to another, this methodology now requires implementation across multiple sites to understand differences in transformation of data and outcome measuring.

## Background

Health care systems globally are under ever increasing pressures to sustain services that continue to meet rising demands resulting from an ageing and expanding population, against the constraints of stagnant financial resources.

Within the last two decades, there has been a change in focus in the delivery methods of healthcare towards the idea of increasing ‘value’ and the concept of Value-Based Health Care (VBHC) in comparison to the traditional Fee For Service (FFS) approach. VBHC is based on the research of Professor Michael Porter in the United States (US), where the fundamental principle is focused on accomplishing increased values for patients by achieving the best outcomes at the lowest cost.^1–3^ By doing so, it allows health services to strive for continuous improvements both for patients; in terms of better outcomes, and the health organization; better outcomes associated with lower costs. Key differences in health service management between the US and United Kingdom (UK), means that VBHC initiatives in the UK and particularly England may differ from Porter’s classic theory.

VBHC as Porter describes, requires a seven stage systematic approach; the first being to organize care around a specific medical condition or closely related set of medical conditions and develop an integrated practice unit (IPU) with appropriately trained staff and facilities. The next two stages measure outcomes and costs for patients with that certain medical condition: outcome measures ideally need to consist of those that are of value to the patient, such as survival, quality of life and holistic needs, alongside those that are of value to the healthcare system; costs are defined as calculating the whole costs of the medical condition, not just restricted to a particular department and over a cycle of care or care pathway. The fourth is to implement an episode based or bundle payment system for a completed care cycle. Stages five and six implement the system integration across a network of facilities including non-acute and community services followed by an expanded geographical area. The final, entails more effective and efficient use of Information systems allowing improved reporting capability and subsequently outcome measuring.

Attempts towards initiating VBHC within the UK have differed somewhat from Porter’s previously described model and need to realise three main functional levels of value: patient; interventional or technical; and population or allocative. With this in mind, we have developed a systematic approach towards adopting a VBHC model in heart failure by collecting retrospective data from the numerous and unlinked information systems within the National Health Service (NHS). We note that, while not necessarily capturing all outcomes that matter to patients as per the Porter’s definition of VBHC (e.g. ability to live an independent life, lifestyle disruption), the collected data provide a comprehensive view of hospital-recorded and medically-related outcomes.

Heart failure is a progressive and chronic condition associated with high morbidity and mortality worldwide. In the UK alone, it is estimated that almost one million people are affected,^4^ although this figure is likely underestimated due to an ageing population. Heart failure in the UK is associated with significant financial burden, accounting for 2% of all NHS inpatient bed days and 2% of its annual expenditure.^4^ Admission rates are expected to rise by as much as 50% over the next 25 years,^4^ suggesting that the current standard of care is not fit for purpose. Could the implementation of a VBHC strategy towards heart failure ensure that the NHS is equipped and ready to meet the present and anticipated future demands?

The UK is reasonably advanced in collecting hard outcome measures such as hospital admissions and mortality through our compulsory national audit, which is now the largest in the world.^4^ Other patient-related outcome measures, however, are less well captured. What has never been developed though, is an automated system to measure accurately both commissioner cost and cost for a patient with heart failure across their whole journey. Without this, true adoption of VBHC into the NHS will not be possible.

In this work, we present a systematic approach to collecting, validating and analysing data from multiple sources and information systems, with the aim of designing and endorsing an automatic system to capture health outcomes data in heart failure to support future VBHC models.

## Methods

**Figure 1.**
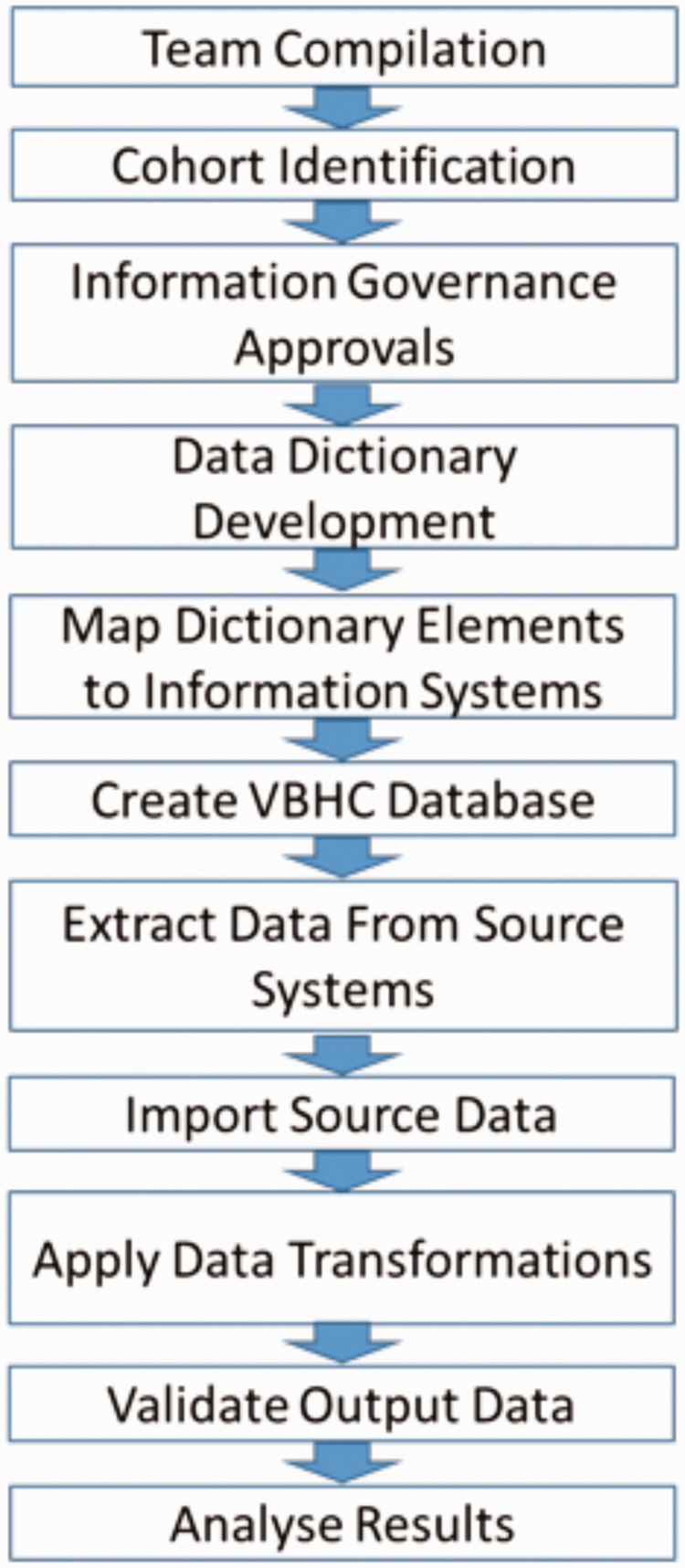
Flow diagram depicting stages of methodology.

The stages of our methodology are summarised in Figure 1, with a more detailed explanation documented below.

### Patient cohort and data access

To ascertain the feasibility of mapping care pathways for heart failure patients, we restricted our cohort to include patients with both a diagnosis of Heart Failure with reduced Ejection Fraction (HFrEF) and Cardiac Implantable Electronic Devices (CIED); either Cardiac Resynchronisation Therapy (CRT) or Implantable Cardioverter Defibrillator (ICD). HFrEF is defined as having estimated or calculated Left Ventricular (LV) ejection fractions of ≤40% on transthoracic echocardiography assessment.^5^ In order to study value of care across a specified geography, we further restricted our cohort to only include patients from the two local geographical areas, based upon clinical care commissioning groups (CCG) to whom Guy’s and St Thomas’ NHS Foundation Trust (GSTT) is the local secondary care provider. The CCG of a patient was determined by the patient’s GP practice at the time of the admission for their device procedure.

A retrospective audit was registered and approved within GSTT, to gain access to relevant patient healthcare data for patients with HFrEF, who underwent an ICD or CRT device related procedure between the 1st of January 2014 and 31st of December 2016. Device procedure within the three year period was identified from the trust dataset required for upload to the National Audit of Cardiac Rhythm Management (CRM). This audit, established in 2014, collects mandatory data on every CIED related procedure from all healthcare centres across the UK. Not all patients with ICDs in-situ have HFrEF, so patients who underwent an ICD device procedure, had their LV ejection fractions manually checked against echocardiogram reports to ensure they met criteria for inclusion.

### Data gathering

We developed a data dictionary of items relevant to VBHC in heart failure. In total, over one hundred and fifty separate data elements were collected for each patient, including general demographic data, relevant history of comorbidities, medications and routine investigations including haematological and biochemical laboratory testing, radiology and cardiac testing. Key to developing any VBHC system, we needed to determine specific ‘outcome' measures. Mortality was documented as standard. Patient valued outcomes included symptom status, using the New York Heart Association (NYHA) class, the six-minute walk test and quality of life measures such as the Minnesota Living with Heart Failure Questionnaire. These outcome measures were informed by, but not exhaustive of the International Consortium for Health Outcome Measurements (ICHOM) Heart Failure Reference Guide.^6^ Health system valued outcomes were focused on health care utilisation (as this is where most costs are attributed) and were inclusive of inpatient admissions; both non-elective and elective, outpatient department (OPD) visits, Accident and Emergency (A&E) attendances and community appointments. For feasibility purposes, we selected a twenty-four month period (12 months pre and post-CIED procedure) to record health care utilisation. As costs are fundamental in developing VBHC, extra care was taken to ensure that financial data was as accurate and complete as possible. Our costing methodology used patient level individual costings rather than reference or tariff costings. We opted to do this as, costs relate to actual expenditure by the Trust whereas tariffs relate to reimbursement to the Trust for delivery of a specific service and reference costs are the average cost to the NHS for providing a defined service in a given financial year. The NHS has a National tariff of prices and rules used by commissioners and care providers to reimburse Trusts. In respect to inpatient admissions, rather than using reference costs, we have obtained detailed patient-level and unit costings from GSTT Finance. This is inclusive of GSTT calculated costs related to resource use such as devices, staffing, estate overheads and bed-days, along with commissioner cost data, broadly based on nationally agreed tariffs which are used to charge the commissioners. Missing costs data was observed for 10% of inpatient admissions and 7% of OPD visits. For missing inpatient costs we used the predicted values from a multi-variable linear regression model which included bed-days, critical care days, type of admission and whether a CIED procedure was performed during the admission. We added some random noise to the imputed values (with a standard deviation equal to the root-mean-square error), to give some variability around the regression line. For the majority of the missing outpatient data we used reference costs and where this wasn’t possible, we simply used median costs.

Data dictionary items were then matched to the relevant information system from where that data could be sourced and extracted (see Table 1). UK NHS Trusts capture a wealth of digital healthcare data on their patients, but it may be spread across multiple disconnected database management systems whose functionality is specific to their purpose. It is therefore not trivial to organise and integrate these datasets in a way that supports policy and improves decision making. To facilitate integrated patient reporting from these isolated systems we created a purpose-built VBHC Database that receives the data from the source systems, transforms it into predefined outputs and loads it into a relational database structure ready for bespoke interrogation and integrated management reporting. Data gathering was predominantly automated and could be queried and extracted directly from hospital systems although occasionally this required a specific request to authorised personnel within a relevant hospital department, e.g. finance or via an external database such as Office of National Statistics (ONS) or National Institute for Cardiovascular Outcomes Research (NICOR). Any remaining data items were extracted via a manual process of reading the patient healthcare record. Table 2 illustrates the proportion of data extracts that were automated or manual. We should highlight that the majority of those pulled manually, could be automated by more effective use of existing information systems.

**Table 1.** Mapping of data elements to source systems.

Data element	Source system(s)	Extraction method
Demographics	PIMS, NICOR	Automatic
Diagnoses and Comorbidities	Multiple Systems via Trust Data Warehouse	Automatic
Radiology	EPR	Manual
Cardiac Investigations	TOMCAT	Automatic and Manual
Haematological and Biochemical Laboratory Testing	EPR	Automatic
Surgery and Procedures	Multiple Systems via Trust Data Warehouse	Automatic
Medications	LCR, JAC PHARMACY	Automatic and Manual
Healthcare Utilisations	Multiple Systems via Trust Data Warehouse, CARE NOTES	Automatic
Patient Reported Outcomes	EPR	Manual
Mortality	ONS	Automatic

Abbreviations: PIMS: Profile Information Management System; NICOR: National Institute for Cardiovascular Outcomes Research; EPR: Electronic Patient Record; TOMCAT: a purpose-built systems database to hold details on cardiological investigations; LCR: Local Care Record; JAC: Justice Administration Commissions; ONS: Office for National Statistics.

**Table 2.** Breakdown of the data extraction method.

Extraction Method	Percentage of data elements
Automatic	70%
Manual	30%

### Integration and transformation

As the various data items were obtained from multiple information systems, importing data into the purpose-built VBHC database required a systematic and regulated approach. Firstly, extracts required matching of the patient identifier, most commonly a unique patient hospital ID number or NHS number, the latter particularly, when extracting and importing primary care data. Secondly, some data required a degree of transformation, converting source data to a more desirable output format, with detailed documentation of all steps of data processing and transformation stages encoded in a Standard Operating Procedures (SOP) for audit and traceability purposes.

All of our diagnostic and procedural data were derived from medical coding. For comorbidity identification and primary admission diagnosis from inpatient admissions, the International Classification of Diseases Version 10 (ICD-10) diagnosis codes were used from the patient hospital record. For surgical and procedural events we used Office of Population Censuses and Surveys Version 4 (OPCS-4) procedure codes from the patient record. The decision to use ICD diagnosis codes was two-fold; primarily, they are internationally recognised and validated which will aid the expansion and up-scaling of VBHC, and secondly, because ICD codes contribute to the calculated commissioner cost for any inpatient admission within the NHS. Essentially, UK hospitals are obliged to perform accurate coding in order to calculate precise costs to the NHS for its utilization.

Data transformation, however, did highlight some important features in UK healthcare data; for example, a single period in the hospital as an inpatient can be represented as multiple episodes in the inpatient reporting system. In addition, single periods as an inpatient can be characterized by multiple primary diagnoses. As a major part of our measured outcomes included inpatient admissions, we wanted to evaluate this data at a higher level of detail, including specifying if the admission was heart failure related, cardiac related or non-cardiac based on the ICD diagnosis code. To achieve this, it was necessary to first group hospital episodes and spells so that they are represented by a single record. Within the UK, a hospital admission, or spell, refers to the continuous period of time that the patient is within the trust, which can include more than one episode. An episode refers to the time spent under the care of an individual consultant. Each grouped hospital single record was then processed by grouped admission ICD diagnosis codes to prioritise heart failure related diagnoses over other cardiac diagnoses over non-cardiac diagnoses. Appropriate and stringent validation of source data, as described below, was necessary to provide assurance that data was interpreted appropriately.

### Validation and quality assessment

Effective validation of the output data was crucial to assess the quality of the source data and to identify any issues arising from the data transformation. We developed validation strategies for each of the data elements, which varied based upon several considerations including; the data source, the method of extraction, the complexity of transformation and the importance of data elements to analysis (cost, mortality, healthcare utilisation and patient outcomes being crucial in assessing value).

An initial basic assessment of quality was made on all output data items, and documented in the data dictionary with comments and a Red-Amber-Green (RAG) risk rating (see Table 3). The basic assessment evaluated availability; which included the ease of obtaining data, the extraction method, percentage completeness, range of values and other relevant factors such as the degree of transformation needed or integrating data across multiple sources.

**Table 3.** RAG results following initial basic validation assessment.

RAG rating	No. of data elements	Percentage
R	16	10.3%
A	72	46.5%
G	67	43.2%

More comprehensive checks and validations were completed on specific data elements where considered appropriate, based on the considerations above and the results of the basic assessment. Random sampling of a percentage of the patient cohort was performed and data reconciled against the source system, an alternative hospital system or against clinical expectations and comparison of incidence of results against an external dataset such as NICOR or ONS (see Table 4).

**Table 4.** Example results following comprehensive validation assessment.

Data element	Complete-ness	Range as expected?	Random sample reconciled against source system?	Random sample reconciled against separate GSTT dataset	Reconciled against external dataset	Conclusions	RAG
COMORBIDITY	100	Yes	Not applicable	Yes. 97% match	Not applicable	Our investigations suggest that this data is of good quality.	G
INPATIENT UTILISATION DATA	100	Yes	Yes. 100% match	Not applicable	Not applicable	Our investigations suggest that this data is of good quality.	G
NYHA SYMPTOM CLASS	96	Yes	Yes, 50% match	Not applicable	Not applicable	Clinical interpretation led to a low correlation (consistent with low inter-observer reliability)	R
DATE OF DEATH	100	Yes	Yes 100% match	Not applicable	Yes, 85% match.	Delays occur updating GSTT source. Decision made to use external data as source (ONS)	G

NYHA: New York Heart Association.

Any mismatch between the original data values and the validation check values were relayed to the relevant member of the team to perform further checks on the data. Further analysis would then either confirm a validity issue or highlight the reason which would explain anomalies in data values. All checks undertaken were documented in a database documentation and included details of any clinical or technical limitations identified. A more detailed description of our methodology is documented in our Standard Operating Procedure (SOP) which is supplied as an online technical appendix.

While the process from devising to implementing our methodology has required significant time resource and informatics expertise to produce a robust and validated dataset, the methodology can now be efficiently scaled up to any sized cohort within our trust.

## Results

One hundred and thirty-six ICD and CRT related procedures were performed on 134 patients between 1st January 2014 and 31st December 2016 (Table 5). Two patients underwent 2 separate procedures as a result of upgrade from ICD to CRT-D in the 3-year timeframe therefore accounting for 136 procedures.

**Table 5.** CIED procedures between 1 January 2014 and 31 December 2016 in patients with HFrEF, within the pre-defined restricted GSTT geographical location.

	ICD	CRT-P	CRT-D	Total
New Implant	22	17	38	77
Upgrade	0	14	8	22
Box Change	4	7	26	37
TOTAL	26	38	72	136

The limited number of subjects within this cohort limits the generalisability of complex findings and statistical analysis, however, by analysing outliers and individual pathways, we can begin to identify important themes related to outcome and value.

**Figure 2.**
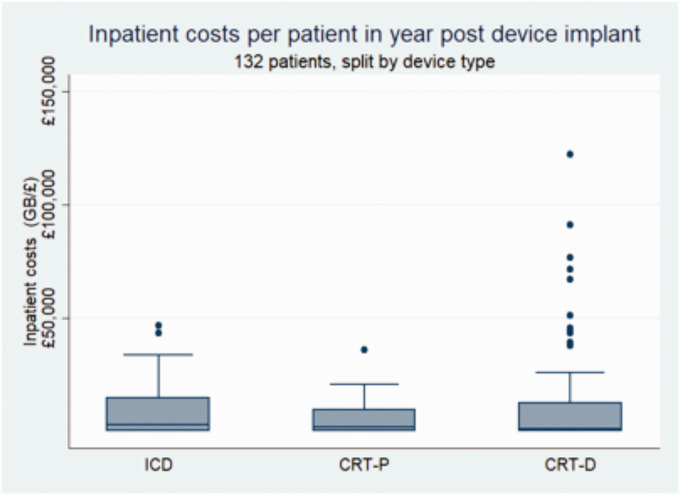
Box plot depicting the inpatient costs in the one year post device procedure by device type.

**Figure 3.**
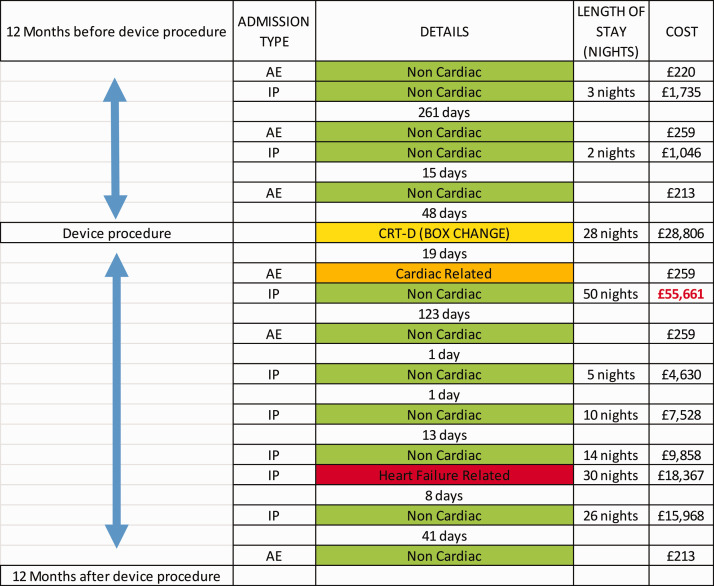
24-month inpatient pathway (defined by the period of 12 months pre and post device procedure) for CRT-D device infection outlier. Colour key code: green = non-cardiac related, amber = cardiac or cardiovascular-related, and red = heart failure related admission. Yellow = CIED procedure. AE = Accident and Emergency attendance. IP = Inpatient admission.

The need for an outlier analysis was highlighted when we calculated actual inpatient costs in the 12 month period post device procedure. Figure 2 shows the variation in costs between the three different types of complex devices in the one-year post device implant. There are a cluster of outliers in the CRT-D group associated with high levels of costs, which were analysed in more detail at an individual level. The inpatient costs in the four leading outliers were attributed to device-related complications, three of which were infections requiring prolonged admission, device explant and subsequent re-implant before discharge. We have plotted the inpatient admissions journey for one of these outliers over a 24 month period pre and post device procedure with documented associated inpatient costs (Figure 3). The most expensive admission costing (£55,661) relates to the admission caused by a device infection.

**Figure 4.**
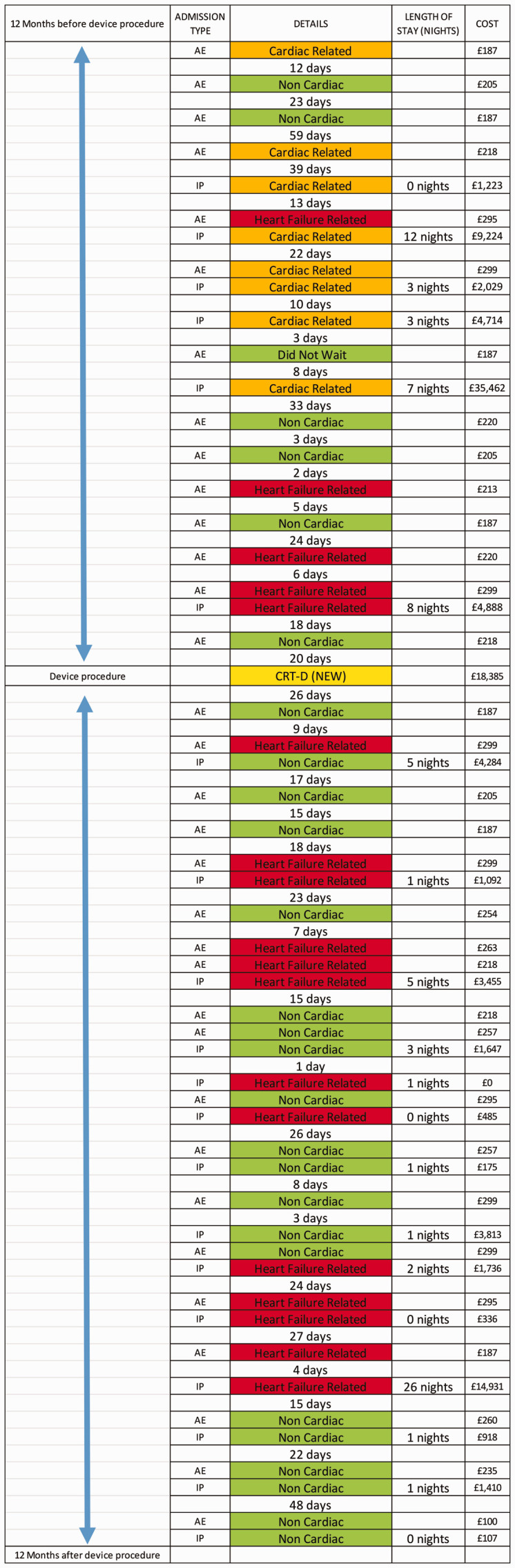
Plotted 24 month patient pathway showing A&E attendances, inpatient admissions and inpatient costs in a patient with 11 listed comorbidities including anxiety mood disorder. Colour key code: green = non-cardiac related, amber = cardiac or cardiovascular -related, and red = heart failure related admission. Yellow = CIED procedure. AE = Accident and Emergency attendance. IP = Inpatient admission.

Another important theme highlighted from rudimentary analysis is the significant number of associated comorbidities in patients with HFrEF and CIEDs. This potentially highlights the need for holistic care by directing attention to common target comorbidities associated with increased healthcare utilisation and higher costs. Table 6 shows a relationship between annual inpatient costs and comorbidity counts and Figure 4 illustrates an example of a patient in our cohort with 11 comorbidities and a total of 33 A&E attendances and inpatient admissions captured in the 12 month periods pre and post device procedure.

**Table 6.** Inpatient costs in the 12 months post device procedure grouped by comorbidity count.

No. comorbidities	Mean cost per patient	Median cost per patient
0–2	£2,410	£0
3–5	£11,123	£1,854
6+	£16,297	£6,657

Our, now purpose built database allows for systematic approach to analysis, which can determine both health service related and patient related outcomes for a said independent variable or intervention, as exampled in Table 8.Our group has recently published work using this database on a said intervention and its outcomes on CIED infection related healthcare costs confirming its functionality.^7^ As previously mentioned, the limitation in our small cohort does not allow for meaningful statistical analysis, however, if the population was upscaled nationally or widened to include all heart failure populations, our automated system would be able to produce data that could guide CCG decisions on NHS expenditure.

**Table 8.** Example of analysis of variable or intervention on health care service and patient related outcomes to aid ‘value’ assessment.

			Average emergency admissions in 12 months post implant			Average emergency bed-days in 12 months post implant			Average elective admissions in 12 months post implant			Average OP appointments in 12 months post implant		Average A&E visits post implant	Average of PROM 1	Average of PROM 2	Average cost in 12 months post implant		Average commissioner costs in 12 months post implant	
Variable A	Total Procedures	Average age	Heart Failure	Cardiac (non-HF)	Non-cardiac	Heart Failure	Cardiac (non-HF)	Non-cardiac	Heart Failure	Cardiac (non-HF)	Non-cardiac	Cardiac	Non-cardiac		NYHA	6MWT	In-patient	Outpatient	In-patient	Outpatient
Response X	89	70.0	0.3	0.1	0.7	3.5	0.6	5.4	0.1	0.1	0.6	6.6	14.4	1.7	1.9	343	£10,737	£2,451	£7,416	£1,913
Response Y	41	73.4	0.0	0.0	0.5	0.5	0.4	4.4	0.0	0.0	0.4	4.0	11.1	1.0	1.8	481	£5,538	£1,528	£4,101	£1,259
Response Z	6	65.0	0.3	0.0	0.8	5.3	0.0	13.7	0.2	0.0	0.7	3.3	4.3	1.5	2.2		£16,676	£918	£9,743	£727

PROM: Patient related Outcome Measure; NYHA: New York Heart Association; 6MWT: Six Minute Walk Test.

Overall, hospital related outsome measures derived from automated data within NHS IT systems were reliable, robust and complete, however patient related outcome measures where less reliably complete and required a more manual data extraction. For example, measures of patient quality of life such as the six-minute walk test and the Minnesota Living with Heart Failure questionnaire which were only available for 15% of patients.

## Limitations

The central challenge surrounding VBHC is the selection of proper short to mid-term patient relevant outcomes and how those outcomes are connected with hard outcomes such as survival. It is possible that two different interventions could result in identical efficacy in terms of short to mid-term patient relevant outcomes, yet result in significant and substantial differences in longer term outcomes. This has the potential to mislead commissioners. Presently, the authors do not have proper solution to address this limitations other than to emphasise that more research is required to add to the understanding of the potential role VBHC could have in modern health care setting. Additionally when analysing data from a number of non-randomised sources, as would be required using a VBHC strategy, the presence of bias needs accounting for during analysis. Any VBHC model would require a strategy to address this, possibly through the use of direct acrylic graphs or single worls intervention graphs, although due to the small sample size of of this work, this was not addressed in our own individual piece of work but would be an essential requirement for an future and upscaled work. It is important to highlight that this work collected retrospective data and thus bears the inherent limitations of such data, chiefly being that we were limited to working with records and information that had been collected for an entirely different purpose. Consequently, several limitations were identified during data collection and processing. Some of this relates to staff compliance and use of information systems and a lack of UK data standards to ensure and enforce capture of data elements. For certain key elements, this led to having to manually extract data, which is prone to subjectivity and error, and is significantly more time-consuming. Another example includes data elements related to patient quality of life measures such as the six-minute walk test and the Minnesota Living with Heart Failure questionnaire which were only available for 15% of patients. Explanations for this include the retrospective data collection method but also patient participation and compliance; the heart failure service within GSTT diligently post questionnaires to individuals, however, most do not complete and respond. It also highlights a need for improved collection of this outcome data from members of the heart failure team when assessing heart failure patients, which needs addressing through improved education and training of relevant staff and is likely to be more reliably performed during a face-to-face encounter with the patient. Finally, NHS information systems are usually multiple and not always linked or integrated to one another, increasing the time and effort for data extraction and any ongoing processing and transformation.

## Discussion

Effective use of value-based health care across geographies, rather than institutions, is one of the key long term visions of the NHS.^8^ Measurement of outcomes, costs and outcome-related patient characteristics is integral to VBHC. In this manuscript, we have described the methodology involved in creating the infrastructure necessary to have meaningful and accurate data in real time that can help inform commissioning. As a proof of concept, we have purposefully attempted to develop a system that can integrate disparate information systems so that it can be scalable across both other conditions and also geographies. We also felt it was important to design a system that measured both costs and commissioner cost across a sector, as both are needed to inform the best way to improve a whole system.

Given the lack of complete data in patient valued outcome measures, the focus of any future analysis of this data will be heavily weighted towards health care system value-based outcomes. However, we feel this can be overcome by using a prospective model and allowing for improvements in collection of quality of life and patient related outcome measures. Acquisition of comprehensive data inclusive of primary care, as well as secondary care, is vital to perform VBHC, as any costing outcomes must include the ‘entire’ cost of a medical condition to the NHS. Whilst data capturing health care utilisation in secondary care establishments is robust and highly detailed, primary care data is more challenging to acquire, particularly through direct attempts using hospital information systems. Although within GSTT Trust and the local CCG, this issue will not be of concern, due to the initiation of a ‘Local Care Record’ which combines patient data from secondary care and primary care and is accessible to clinical staff within the Trust, we appreciate that up-scaling this methodology to other geographical areas may pose challenges.

It is important to emphasise the different types of financial data that we have accessed. The majority of published work in this area uses financial data derived from reference costs and is therefore not an accurate reflection of expenditure. We have managed to obtain both trust costs and commissioner cost at an individual patient transaction level for any inpatient related stay. Costs represent the entire outlay of the hospital stay, and covers everything from device to staffing, overhead and diagnostic testing. Commissioner cost, on the other hand, is based on nationally and locally agreed reimbursement tariffs. Table 7 highlights the variation between these two types of costs when calculated per device group and at a patient level in the twelve months post device fitting. Contrary to many studies, we have collected highly detailed costing data relating to patient health care utilization. Unlike previous work, which uses financial data derived from reference costs, our inpatient admission, A&E and OPD department related financial data was derived from patient level costing and represent the actual cost to the Trust. Inpatient admission costs represented the entire outlay of the hospital stay, including device, staffing, overheads and diagnostic testing. We can also record hospital commissioner cost for patient care, based on nationally and locally agreed reimbursement tariffs. Community Heart Failure Nurse visit costs are separately commissioned and available to the Trust drawn from reference costings. Overall, community-related costs contribute less significantly to the overall 24-month care pathway surrounding CIED procedures in contrast to inpatient admissions.

**Table 7.** Differences between inpatient cost and commissioner cost by device type in the 12 months post device procedure.

Device type	Cost	Commissioner cost	No patients
CRT-D	£8,93,485	£589,496	72
CRT-P	£1,87,354	£161,809	38
ICD	£2,29,760	£152,364	26

CRT-D: Cardiac Resynchonisation Therapy + Defibrillator; CRT-P: Cardiac Resynchronisation Therapy Pacemaker; ICD: Implantable Cardioverter Defibrillator.

Initial and rudimentary analysis within our cohort has demonstrated increased healthcare costs amongst HFrEF patients with multiple co-morbidities, however further work is required to identify certain key co-morbidities that are associated with the highest non-elective admissions and subsequent costs in order to re-structure management pathways and improve multi-disciplinary team working amongst varying sub-speciality medical professionals. Our results have also highlighted that HFrEF patients with defibrillator therapy adjunct to their CIEDs have accompanying increased health care economic burden; some of which may be explained by CIED related complications, chiefly infection, calling for a review of current CIED procedural protocols to minimise this risk within this particular ‘high risk’ group of patients but also referring to our previous sentence; the prevalence of multiple co-morbidities needs further assessment in determining those patients who may get limited benefit/lower value from defibrillator therapy, to facilitate improved decision making and to optimise pathways and guidelines.

The methodology described here is specific to the information systems and clinical pathways within one London NHS Trust. As it stands, the integration database developed has been customised to the conditions within our Trust, and would, therefore, have to be reworked in order to achieve scalability in differing Trusts electronic healthcare records (EHRs) and pathways. Scalability will be enhanced by ongoing efforts to harmonise and link EHRs, to facilitate a future where standardised systems allow for predictions to be made across hospitals and regions.

We have identified that some key patient valued outcome measures are not always well captured, which is fundamental to implementing VBHC. We believe this is a situation not unique to out Trust and likely to represent a nationwide and global problem, calling for improvements in compliance in collection of patient-related data, which could be resolved nationally by including these measurements as a compulsory standard measure in our national audit or another purpose built automated system. In the future, UK NHS expenditure determined by CCG’s will be increasingly focused towards patient valued or related outcomes and therefore employing standardised patient centred outcomes such as those reported by ICHOM would allow for easier global adoption of VBHC. We acknowledge that individual hospitals maintain local procedures which may deviate from national guidelines and therefore costs and outcome measures may also differ, as a result of variations in procedures and measures across differing NHS Trusts. Our next step is to adapt and develop our methodology across multiple trusts in order to quantify these differences.

## Conclusion

Future healthcare delivery methods and decisions on expenditure will be increasingly based on VBHC models. To achieve this in current practise, we need more effective and efficient use of information systems allowing improved reporting capability and subsequently outcome measuring, as highlighted by the work we have undertaken and presented. Furthermore, key to VBHC principles, improvements are needed in both focusing on and capturing patient related outcome measures to guide commissioners spending. Our work is the first stage towards creating an automated system capable of being utilised for evaluating VBHC in any specified medical condition and on a national or global scale. The next stages of our work includes expanding our cohort to include all heart failure patients within our own Trust and secondly to replicate our methodology in a neighbouring but independent Trust to ascertain its feasibility for expansion and upscale.

## Abbreviations

A&E: Accident and Emergency; CCG: Clinical care Commissioning Group; CIED: Cardiac Implantable Electronic Device; CRM: Cardiac Rhythm Management; CRT: Cardiac Resynchronisation Therapy; EHR: Electronic Health Record; EPR: Electronic Patient Record; FFS: Fee For Service; GSTT: Guy’s and St Thomas’ NHS Foundation Trust; HFrEF: Heart Failure with reduced Ejection Fraction; ICD: Implantable Cardioverter Defibrillator; ICD-10: International Classification of Diseases-Version 10; ICHOM: International Consortium for Health Outcome Measurements; IP: In-Patient; IPU: Integrated Practice Unit; JAC: Justice Administration Commissions; LCR: Local Care Record; LV: Left Ventricular; NHS: National Health Service; NICOR: National Institute for Cardiovascular Outcomes Research; NYHA: New York Heart Association; ONS: Office for National Statistics; OPCS-4: Office of Population Censuses and Surveys-Version 4; OPD: Out Patient Department; PIMS: Profile Information Management System; PROM: Patient Related Outcome Measure; RAG: Red-Amber-Green; SOP: Standard Operating Procedure; 6MWT: Six Minute Walk Test; UK: United Kingdom; US: United States; VBHC: Value-Based Health Care.

## Availability of data

As patient consent was not obtained, raw data is not available on demand to share with the journal.

## Ethics

Ethical approval was not sought for this work. As the work was specific to a single National Health Service Foundation Trust including healthcare service utilisation from a retrospective period, this project was felt to meet criteria for Audit or Service Improvement and appropriate Trust information governance approvals were obtained.

## Supplemental Material

sj-pdf-1-hsm-10.1177_0951484820971442 - Supplemental material for A systematic approach towards implementing value-based health care in heart failure: Understandings from retrospective analysis methods in South London Click here for additional data file. Supplemental material, sj-pdf-1-hsm-10.1177_0951484820971442 for A systematic approach towards implementing value-based health care in heart failure: Understandings from retrospective analysis methods in South London by Emma Burnhope, Michael Waring, Andrew Guilder, Bharti Malhotra, Jorge M Cardoso, Reza Razavi and Gerald Carr-White in Health Services Management Research
