# Supplementary material for: A systematic approach towards implementing value-based health care in heart failure: Understandings from retrospective analysis methods in South London
Source: Health Serv Manage Res. 2020 Nov 30;35(1):37–47. doi: 10.1177/0951484820971442 (PMC8795220; doi:10.1177/0951484820971442)
Supplement: sj-pdf-1-hsm-10.1177_0951484820971442 - Supplemental material for A systematic approach towards implementing value-based health care in heart failure: Understandings from retrospective analysis methods in South London [file sj-pdf-1-hsm-10.1177_0951484820971442.pdf]

Technical Appendix

In support of:
A systematic approach towards implementing Value Based Healthcare in Heart Failure: Understandings from retrospective analysis methods in South London.

Table of Contents

Table of Contents 1

Aim 2

Introduction 2

Planning and Preparation 3

Data Gathering 4

Data Integration and Transformation 6

- Standardisation of output data 6
- Scalability 7
- Traceability 7
- Single Value elements 7
- Multi-value elements 8
- Specific transformation considerations for the Inpatient dataset 9

Data Validation and Quality Assessment 10

- Comprehensive validation checks 11
- Validation Conclusions 12
- Scalable standards compliant validation 12

Aim

The purpose of this procedure is to provide clear standardised guidance on accessing, extracting, categorising, validating and analysing data relating to Heart Failure patients in an NHS Trust

Introduction

UK NHS Trust hospital data management systems are often deployed on local network clusters that often are not fully integrated. In order to be able to analyse patient healthcare utilisations and evaluate them in context of outcomes that are important to patients and costs to a Trust, it is necessary to gather and integrate this data into a single system. This procedure outlines the actions required to extract, transform and load data related to patient transactions and outcomes, together with data on associated income and costs, into a format which is standard and reportable. The development of integrated reports should provide the data framework required to help facilitate the application of Value Based Healthcare in Heart Failure patients.

Planning and Preparation

A project manager will be appointed by the project board to lead the project team and be the primary liaison with the wider team.

The project board will identify or appoint key personnel at the Trust site, this would normally include some or all of the following:

- Chief Investigator
- Co-investigator
- Cardiology research fellow
- Trust data analyst
- Statistical lead
- Database manager

The project manager will arrange a meeting/call with all key stakeholders to introduce the project and begin compiling a project plan.

The Chief Investigator will work with the research fellow and the project partners to develop a dictionary of data points which they believe to be of relevance to the Heart Failure care pathway. This may include investigation of existing standard data sets used in Heart Failure (such as NICOR) and those used to measure patient outcomes (such as ICHOM). Review of the data dictionary by the appointed statistical lead should take place as early as is practicable to ensure all relevant data elements are included on the dictionary prior to data gathering. The dictionary will then be updated. Care should also be taken to include dates of tests, images etc.

The research fellow and team will contact the Trust's Research Governance team to enquire about the specific procedure for registering the project and requesting the data.

Before submitting the proposal to the Research and Development team, the research fellow will consider where the extracted data will be collated, processed and analysed. If the proposed destination for the data is outside of the local Trust's local IT network, this must be specified explicitly in the proposal. Note that data should be anonymised before being securely transferred out of the Trust IT network, and all external data transfers require written approval from the Trust Research and Development office.

The research fellow will submit the proposal to the Trust Research and Development office. The proposal must be approved prior to extraction of any data, and the approval notification should be circulated to the team once provided.

The project manager or Trust analyst will request that the Trust IT helpdesk provide access to a secure working area on the Trust network to gather & store data extracts, and consolidate these into an integrated heart failure database. Any member of the team who will be working directly with the data (research fellow, analyst, data manager) will need to be able to access this shared network resource, so requests for individuals' access should be made at the same time.

The Trust analyst will work with the project manager and the Trust IT helpdesk to ensure that he or she is able to run appropriate statistical software for analysing data within the project working area of the secure Trust network. If the Trust does not offer such software directly on the secure network the Trust analyst may need to request a Trust laptop with the required software installed. The project manager will draft the project plan based on discussions with all key stakeholders and circulate for review.

Data Gathering

The research fellow will meet with the Trust's Head of Heart Failure to define the inclusion criteria of the relevant heart failure patient cohort.

The research fellow will use the local Trust Cardiology data management system (e.g, Guy's and St. Thomas' NHS Foundation trust (GSTT) uses TOMCAT at the time of writing) and the above mentioned inclusion criteria (EF < 40%, Complex device fitted between Jan 2014-Dec 2016) to identify a cohort of patients, and extract hospital patient identifiers and device dates. Note that the criteria may be expanded in future.

The research fellow will save the list of patient hospital identifiers and device dates on the defined secure folder of the Trust network which was set up for the VBHC project.

The Cardiology research fellow and Trust analyst will meet and review each element in the VBHC approved data dictionary, identify the local hospital system and data field which is the source of that data element, and update the data dictionary "Hospital System" column with the agreed source. There may be some output data points which are derived from multiple source data points (e.g. Comorbidities identified from interrogating multiple inpatient diagnosis codes from multiple data points). In such situations where the relationship between source and output data is not straightforward, the full list of source fields should be indicated on the data dictionary, and the method of deriving the output should be detailed to ensure a standardised approach when working across multiple trusts and datasets.

Once all fields on the data dictionary have a hospital system assigned, the Trust analyst will build a table of all required raw data extracts. The output table of required data extracts will include:

- Unique code for each raw data extract (E.g. A01, A02 for automated and M01, M02 for manual data extracts)
- Description of dataset
- Extract source
- Method of extraction
- Summary of filter criteria
- RAG (Red amber green traffic light system) rating for availability and any other risks identified
- Notes of any other pertinent information related to defined dataset

Where relevant, data warehouses should be utilised as an integrated source of patient data. Where data can only be sourced from notes or letters on the Electronic Patient Record (rather than a discrete data field), manual extraction may be needed. This should be considered when building the table of required raw data extracts.

The Trust analyst and study team will use the completed list of required data extracts and knowledge of Trust IT architecture to construct a high level schematic of relevant hospital systems architecture.

The Trust analyst will use the completed list of required data extracts to request and acquire all automated data extracts, updating the list where necessary, noting any issues and adjusting RAG ratings accordingly. These extracts will be stored in the secure area of the Trust network defined for the project. Note that in order to capture all relevant data, it will be necessary to define the period of interest and include data from that period of time prior to the start of the date range for the patient cohort, and the same period after the end of the date range. This will

ensure that the entire period of interest (e.g. one year either side of the device implant date) is covered in the extracts. To ensure data completeness, and to allow for like-for-like comparison of data, consideration should be given to any data items where there may be a lag between date of occurrence and date of reporting (e.g. date of death) and where data is likely to update over time (e.g. mortality). Note that the date range of interest may be expanded in future iterations.

Once an automated extract has been received by the Trust analyst, they will map the data elements on the data dictionary to the data fields on the automated extracts. Where elements can be mapped with confidence to automated data extracts, the columns "Data Extract" and "Extract Field Name" on the data dictionary will be updated with the relevant information and the "Method of extraction" field will be updated to "Automatic". The “Filename” field on the table of data extracts will be updated to reflect the filename of the source data file received. Time should be invested at this point to maximise the automation of data extracts where possible. This may involve discussion with specialist users of specific database applications and requests to specific service providers (e.g. cardiology, hospital pharmacy, Trust informatics, Trust finance) where data is not available within a reporting warehouse.

The research fellow will use the patient cohort list (extract A01) and the list of manual extracts (M01, M02 etc) as the basis for manual extraction of all remaining data elements from source hospital systems. A data file should be created on the secure network drive for each manual extract, containing the patient IDs and the fields identified in the data dictionary. Field labels and value codes should match those of the data dictionary to avoid confusion and misinterpretation. In some situations the research fellow will need to extract multiple data items per data field. Where this relates to one measure pre device implant and one measure post device implant the field names should be prefixed with PRE_ and POST_. In these scenarios it is important to also record the date of capture of the measure.

Once a manual extract is completed the research fellow should notify the Trust analyst that the manual extract is complete and confirm the names of the completed extract files on the secure network. Upon receipt of the notification the Trust analyst will update the data dictionary fields "Method of Extraction", "Data Extract", "Extract Field Name" for the manually extracted data fields.

Data Integration and Transformation

The Trust analyst will check that all of the mappings have been documented on the data dictionary, and will then take a copy of the source data extracts and place them in a working folder. He/she will perform a visual check on the raw data extracts to ensure that the files are in the expected format and that required data fields are populated with the expected type of data. Note that it may be necessary to perform modifications to the data extracts in order to import them successfully into the database. Typically each record within a data extract will need to contain a patient identifier such as hospital ID or NHS number in order to be processed. Where there are multiple data extracts to be imported into a single database table, the analyst must ensure that all column names within each extract are matching prior to import. Any modifications undertaken to allow for importing should be documented in the “Integration Step” columns of the ‘Raw data extracts’ documentation.

Once the Trust analyst is satisfied that the raw data extracts match their expectations, they will import each raw data file into the VBHC dedicated database. The naming convention for the tables should be clearly defined. Using a system of documented codes for tables and other database objects should allow a technical user who is unfamiliar with the project to understand how the data is stored by cross referencing between the data documentation and the database tables.

The Trust analyst will document any anomalies encountered when performing the visual check or when importing the data. Anomalies should be documented in the “Integration Step” columns of the “Raw data extracts” table.

Standardisation of output data

Many data output fields will need to be transformed into standard codes equating to the “Value Codes” column on the data dictionary. For example Sex may be stored in the hospital system as M, F or Male, Female, but the expected output as per the approved data dictionary, is 2 for a Male and 1 for a Female. For each field, where a transformation is required, the Trust analyst will perform a “group by” SQL query on the individual source data field, which will return a complete list of unique responses that have been attributed to that data point within the dataset. Where individual responses can be matched exactly to the output value codes defined in the data dictionary, the Trust analyst will either write a function to convert the outputs to dictionary standardised outputs, or construct a lookup table with the complete list of unique responses and their associated output value code. Lookup tables are more suitable where exact matches can be made between source and output data, and there are a large number of expected values related to that element. Functions are more suitable where matching is done using wildcards (e.g. like operators such as “like ‘C*’” to capture all diagnosis codes related to Cancer), or where there are a small number of possible expected values for an element. This is specifically useful for matching of ranges of [International Classification of Diseases] (ICD) codes and Office of Population Censuses and Surveys (OPCS) Procedure Codes. The Trust analyst must take care when using functions with wildcard matching to ensure that wildcard matches only capture the correct range of values. All such transformations must be documented in the “Intermediate Transformations” documentation, including any functions written and lookup tables used.

Where there is any ambiguity related to the relevance of source data, (e.g. multiple different types of lab tests related to the testing of Creatinine), the Trust analyst will document the full list of responses available in the source data table and request clarification in writing from the research fellow regarding the clinical relevance of each of the responses. The Trust analyst will document the full list of responses and the result of the clarification from the research fellow in the “Data Queries” project documentation.

Lookup tables should follow a defined naming convention.

Scalability

The GSTT database model comprises four distinct parts. The first (tbl1_) relates to the source data tables and is specific to each Trust as it represents the raw extracts obtained from the hospital systems. The second part (tbl2_) relates to the standardised output tables and should be scalable across Trusts as it maps to the standard data dictionary. The third part (tbl3_) is a set of lookup tables which again are very specific to the Trust. The fourth (tbl4) is one or more audit tables which record all changes to data (see traceability heading below).

It is envisaged that in order to make the database model scalable, the output data (tbl2_) should be exported into a standalone database which can be used to hold data on multiple trusts, whereas the other parts (tbl1, tbl3_, tbl4_) are Trust specific so a database would be required for each Trust to hold this data.

Traceability

The Trust analyst must document each transformation of data as it is created. Where functions are used to convert the response values into standard codes, the function should be pasted into the data dictionary in the respective “Transformation specifics” column. Where a lookup table is used, the lookup table should be documented in Lookup tables, and the lookup table name should be pasted into the data dictionary column “Transformation specifics”. Where there are intermediate transformations, these will be documented step by step in the “Intermediate transformations” documentation.

The Trust analyst will set up the VBHC database in such a way that transaction logs are created detailing each action which is applied to the output tables. This provides a complete transaction history of processing of the data and confidence in the validity of the processing applied. SQL triggers may be applied to the output tables to automate the logging of transactions on those tables, and should include the relevant table identifier, field identifier, record identifier, the user identifier, the date and time of the transaction, the old value for a field and the new updated value.

Single Value elements

The Trust analyst will write SQL data queries to isolate and transform the individual data fields. Where there is only one value expected for an element but multiple exist (e.g. Patient Height, DOB should be unique for an individual), the analyst will construct a query to extract *only* those data elements using the table imported from extract *Ax* as the master patient table and using Outer SQL joins to the corresponding table(s) containing the elements to measure. In some cases, SQL joins must be on both hospital ID AND Device date to take into account that an individual patient may have had more than one device implant, and treat these in isolation. The analyst will pull all single value expected elements into the query and group by all elements.

The number of values returned by the query should be equal to the number of device implants (i.e. the number of records in patient master table A1). If the total number of records returned does not equal the number of records in master table A1 then this suggests that a single value field is returning multiple values. In this scenario the Trust analyst will perform a second query on the “single value query” to isolate all patient IDs which have a count greater than one. Close inspection of any patients returning multiple records should highlight where more than one value is being returned by the query for a patient/device implant on a specific data point. If any

such data queries arise, the output dataset should be inserted into a new table. Once the multiple values have been investigated the most relevant value should be agreed on with the team and any other values should be removed. If a large number of expected single value responses return multiple values, the Trust analyst will investigate in more detail the possible causes of the anomaly. Note that where a value is to be captured before and after device implant (e.g. LVEF_TTE), these should be considered as two distinct fields with their PRE and POST prefix and should be treated as distinct elements, with a single value expected for each.

Before writing the single values dataset to an output table, the Trust analyst will edit the query to allow for any transformations of responses to standard value codes. Transformations will be driven by lookup tables or functions as described above. The analyst will also update the field names to correspond to those defined in the data dictionary. The expected output will be a table with a single record per device implant. The Trust analyst will document the transformation process for the single output dataset in the "Intermediate Transformations" document. A set of transformations which provide an output dataset should be labelled appropriately e.g.T01, T02 etc. The queries that make up the transformation should adopt naming conventions such as qry_T01_01, qry_T01_02 where the first indices relate to the reference of the transformation and the second indices _01, _02 relate to the sequential steps of the transformation process.

Multi-value elements

The Trust analyst will then write data queries to transform data fields and records where multiple values may be expected per device implant. In this scenario a separate set of transformations will need to be designed for each group of related data elements. Examples are Inpatient admissions, A&E admissions, Outpatient admissions, Costs, Income, Blood tests etc. For each element group, the Trust analyst will construct queries to isolate only those elements relevant to the group. They will use the table imported from extract Ax as the master table and Outer SQL joins to corresponding tables. Where joining data from multiple tables, joins may need to be made across both hospital ID AND device date on the tables being joined, to isolate data to a device implant where an individual patient may have had more than one device implant. Each device implant may then be processed in isolation. The analyst will apply date filters to ensure that only relevant events are included (For our study this was one year prior to admission for device implant and one year after discharge following device implant). The Trust analyst will design and document a set of transformations for each element group. E.g. Inpatient admissions, A&E admissions etc. Before writing the grouped multiple values datasets to an output table, the Trust analyst will edit the query to allow for any transformations of responses to standard value codes. Transformations will be driven by lookup tables or functions as described in the 'Single Value elements' section above. The analyst will also update the field names to correspond to those defined in the data dictionary. Finally the Trust analyst will insert the output datasets into tables. The naming convention for the output tables should follow a documented naming convention.

Once all data fields have been included in either the single values table or a multi value table related to a group (such as Inpatient admissions), the Trust analyst will review the output tables to ensure that no direct identifiers such as names, addresses etc. have been included in the output tables. The exceptions to this are hospital ID (which is the unique ID which allows the analyst to join the different raw data extracts together), and Date of Birth (which should have been converted to Month of Birth by transformation as per data dictionary, rendering it less-identifiable.

The Trust analyst will fully document each transformation and integration step within the project documentation.

Specific transformation considerations for the Inpatient dataset

Individual patient inpatient periods or "spells" are sometimes characterised by multiple episodes which in turn contain multiple diagnosis and procedure codes. This is useful for obtaining an overall picture of the patient experience but multiple episodes within a patient inpatient period need to be grouped in order to accurately report on inpatient admissions without double counting. This is compounded by the desire to report on whether a particular inpatient admission was related to Heart Failure, Cardiovascular or something unrelated.
In order to process the multiple inpatient episodes within a spell with consistency, the Trust analyst will need to undertake the following steps when processing the data:

- Join the inpatients source data table to the device admissions table using patient ID
- Group on spell number and run primary diagnosis code through Function "HF related" which determines whether diagnosis relates to Heart Failure, Cardiac or Non Cardiac based on ICD codes. Excluding episode number from the query here removes multiple episodes within the same spell with the same primary diagnosis. Spell number should be retained as this relates to a distinct inpatient stay.
- Crosstab the "HF related" field so that the Heart Failure relatedness is split into three fields, populated with spell number
- Pull the fields from the crosstab query and run a function to prioritise Heart Failure diagnosis, passing the three HF related fields from the crosstab. This prioritises HF related diagnoses over CV over non CV as required, to ensure that Heart Failure related diagnoses are always reported as priority where there are multiple primary diagnoses for an inpatient episode.

Specific transformation considerations for the Procedures & Diagnoses datasets

The Trust analyst will identify the total number of diagnosis codes and procedure code data elements in the inpatient dataset structure. In order to interrogate the full list of codes using SQL database queries, the Trust analyst will write SQL union queries which "stack" the codes on top of each other into a list which is more suitable for interrogation by an SQL query. This list of codes in a single column will then be run against a function which isolates specific procedures or co morbidities of interest from the list of codes.

Data Validation and Quality Assessment

Once the data integration and transformation has been completed and fully documented, the Trust analyst will perform a series of validations and quality assessments to ensure that the output data is of a high quality.

The Trust analyst will examine the transaction logs to ensure that they are working correctly and are recording all transformations to the data as documented.

The Trust analyst will work with the research fellow and the research team to devise and implement validation strategies for data elements. Strategies should take into account the following:

- The source of the data and any corporate validation that may have already occurred (e.g. on the Trust data warehouse)
- How the data was obtained (Automatic extract vs Manual transcription)
- Level of transformation applied to the data
- The overall importance of specific data items or data groups to general analysis and reporting (e.g. for Value Based Healthcare, data elements related to cost, income and patient outcomes are crucial in assessing value and therefore should be subject to a higher degree of scrutiny and validation
- The overall importance of specific data items or data groups to any specific research questions being asked

In the first instance, the Trust analyst will make an assessment of the following 'simple to measure' factors and document in the data dictionary:

- Availability (including ease of obtaining data & auto vs manual extraction method),
- % completeness
- Visual check of range of values (sort by field, scan range visually, find min and max, any outliers or unexpected values)
- Any other factor that may impact upon quality such as pre-existing validation, degree of transformation needed, data coming from multiple sources etc.

For single value data elements, data completeness can be calculated by dividing the number of device implants which have a meaningful value for a data element by the total number of device implants in the cohort, to give a percentage. The Trust analyst will populate the data dictionary "% complete" field with the results of this calculation. Some caution needs to be exercised here in relation to missing values. The analyst must take care to consider missing values, which have been converted to missing values codes such as 999. Some single value data elements (such as co-morbidities, which are wholly derived from Trust diagnosis codes) are more challenging to assess in terms of completeness.

Where there is a finite expected range for a data element value, the Trust analyst will perform checks on the range of values in the source and output data. For example, Date of Birth range should be within roughly one hundred years prior to the current date and it is expected that most individuals will lie in the earlier part of this range. The range will be documented on the data dictionary. Post codes would be expected to broadly match the catchment area of the hospital Trust so checks on the range of postcode areas in the dataset should confirm expectations and any outliers should be investigated against patient address and GP address.

Simple RAG risk ratings are to be completed for each field in the Data Dictionary based on evaluation of the above four factors. For example, inability to extract data automatically for a data element would mean an amber rating at best, due to the risks around extracting data manually. This provides us with a basic evaluation of risks for each data element without engaging in more in depth validation checks.

The validation strategy for each data element should then consider the simple RAG risk rating previously mentioned, together with the importance of the data field to the research being undertaken, so we know that for a VBHC study, healthcare utilisations, costs, income and any variables required for answering a research question are extremely important fields to concentrate our validation efforts on.

Where the simple RAG risk rating is green and ranges, completeness and manual checks have not provided any mismatches or cause for concern, it may be considered less imperative to perform more detailed checks against separate internal or external systems in order to have a high degree of confidence in the data.

Where data is critical to the purpose of the reporting function (e.g. Costs, Utilisations etc for VBHC), and/or the Risk RAG is amber or red, (e.g. when more rudimentary checks on data quality give unexpected results or cause for concern), this would indicate a lack of confidence in the data and this should promote a more thorough investigation into data quality.

Comprehensive validation checks

The following checks and validations should be considered on specific data elements where appropriate:

- Randomly sampling a percentage of participants and reconciling data against source system
- Reconciling data against another hospital system or dataset, or against clinical expectations
- Comparison of incidence of results against an external dataset (e.g. NICOR, Office of National Statistics etc)

Where random checks are to be performed on data points, the Trust analyst will use an appropriate function on a suitable application to pick the appropriate number of patient IDs from the full cohort. In the first instance, 10% of patients may be selected at random and checked. Once the verification has been completed, the research fellow will save the results on the secure network and inform the Trust analyst of the location of the validation file. Upon receipt of all the validation files, the Trust analyst will automatically reconcile the data against the original data points for those patients and report on any anomalies. When original data is different from the validation review, it would most likely need a manual assessment which of the values is correct. (as described in next section). It is recommended that a new database is set up for the purposes of validation and reconciliations of data. Each query which validates or reconciles for this purpose should be saved and a reference to the query added to the validation documentation.

The Trust analyst will liaise with the research fellow to isolate any data points which are required for analysis of specific research questions. Where possible, the research fellow will be able to indicate according to their general clinical knowledge and specific understanding of the care pathway, expected values or ranges for certain data points.

Detailed review and checking of data elements as described should assist the Trust analyst understanding any limitations of the data and these should be documented alongside the validation checks. Limitations may be clinical or technical, and these should be documented

separately. For example, NYHA class is subject to clinical interpretation so differences in calculation of the value for a patient between two clinicians is a clinical limitation of the data. If there are issues in obtaining historical or recent costs data, this should be listed as a technical limitation of the data.

Where undertaken, these comprehensive validation checks, together with clinical and technical limitations identified, should be documented in the validation section of the database documentation.

Where appropriate, the Trust analyst will reconcile units between the data dictionary and the hospital datasets to ensure that they match. Where units are found to be different, the Trust analyst will amend the data dictionary and highlight the difference to allow for further investigation if necessary.

Validation Conclusions

The trust analyst will reconcile and document all data element validations in the 'Validations' section of the documentation. Where possible the analyst will complete or request from the research team the range of checks documented in this section of the procedure. Where there is a mismatch between the original data values and the validation check values, the Trust analyst will update the relevant member of the team to perform further manual checks on the data. Further analysis would then either confirm a validity issue or highlight some other reason which would explain anomalies in data values. In each case the case study must be fully documented in the 'Validations' document. Once this has occurred and the team has updated the validation documentation with limitations and conclusions of the validation, they should assign a validation RAG rating to the data element indicating its completeness and reliability.

Scalable standards compliant validation

Health Level-7 (HL7) refers to a set of international standards for the transfer of clinical and administrative data between software applications used by various healthcare providers. Such guidelines or data standards are a set of rules that allow information to be shared and processed in a uniform and consistent manner. These data standards are meant to allow healthcare organizations to easily share clinical information.

FHIR – Fast Healthcare Interoperability Resources is the latest implementation of HL7 standards. The Trust analyst will investigate the Trust hospital systems to ascertain what level of alignment to FHIR HL7 standards exists within the relevant parts of the Patient Healthcare Record.

Where there is a high degree of alignment with FHIR or other HL7 compliant standards (SNOMED, LOINC), the Trust analyst will seek, where possible, to develop an automatic process of data checks based around those standards. The Trust analyst will investigate the possibility that alignment with such standards might provide the scalability that will be needed to implement the checks and validations described above without the need for manual tailoring of such checks which is required when there is no standards compliance in the healthcare systems being interrogated.
